# Association between Diet Quality and Eating Behavior in Type 2 Diabetes Adults: A Cross-Sectional Study

**DOI:** 10.3390/nu16132047

**Published:** 2024-06-27

**Authors:** Ana Maria Gal, Lidia Iuliana Arhire, Andreea Gherasim, Mariana Graur, Otilia Nita, Oana Dumitrascu, Raluca Meda Soimaru, Alina Delia Popa, Laura Mihalache

**Affiliations:** 1 Faculty of Medicine, “Grigore T. Popa” University of Medicine and Pharmacy, 700115 Iasi, Romania; ana-maria.a.ilisei@d.umfiasi.ro (A.M.G.); andreea.gherasim@umfiasi.ro (A.G.); otilia.nita@umfiasi.ro (O.N.); oana.dumitrascu@arcadiamedical.ro (O.D.); raluca-meda-m-frigura@d.umfiasi.ro (R.M.S.); alina.popa@umfiasi.ro (A.D.P.); laura.mihalache@umfiasi.ro (L.M.); 2 Faculty of Medicine and Biological Sciences, Stefan cel Mare University, 720229 Suceava, Romania; mariana.graur@usm.ro

**Keywords:** DQI-I, DEBQ, emotional eating, external eating, restrained eating, dietary behavior

## Abstract

Type 2 diabetes mellitus (T2DM) has become a global epidemic. To effectively control T2DM, individuals must adhere to a high-quality diet that encompasses not only healthy dietary patterns but also promotes positive eating behaviors. We conducted a cross-sectional study on 314 patients with T2DM, and we evaluated the diet quality and also examined the associations between eating behavior, diet quality, and anthropometric and clinical factors in T2DM patients. We used the Diet Quality Index-International and Dutch Eating Behavior Questionnaire to assess dietary characteristics. We found that women had a significantly higher diet quality than men (61.40 vs. 58.68, *p* = 0.002) but were also more prone to emotional eating (2.00 vs. 1.53, *p* < 0.001) and restrained eating (2.39 vs. 2.05, *p* = 0.002). Restrained eating correlated with duration of diabetes (r = −0.169, *p* = 0.003), body mass index (r = 0.182, *p* = 0.001), and external eating with glycated hemoglobin (r = 0.114, *p* = 0.044). Patients with emotional eating had a higher vitamin C adequacy score (β = 0.117, *p* = 0.045). External eating was positively associated with grain adequacy (β = 0.208, *p* < 0.001) and negatively associated with empty-calorie food moderation score (β = −0.125, *p* = 0.032). For restrained eating, we found associations with vitamin C adequacy (β = −0.138, *p* = 0.017) and fruit adequacy (β = 0.125, *p* = 0.033). In conclusion, the results of this study provide valuable insight into dietary behavior and emphasize the importance of promoting healthy eating habits for T2DM patients.

## 1. Introduction

Type 2 diabetes mellitus (T2DM) has emerged as a global epidemic and is the most prevalent form of diabetes [1]. According to the PREDATORR study, the most recent comprehensive national epidemiological assessment of diabetes mellitus (DM) from Romania, the prevalence of DM was 11.6% in 2015 [2]. Research by Gherbon et al. identified the highest prevalence of T2DM in individuals aged 60–69 years, with a prevalence rate of 35% [3]. Additionally, a thorough examination has also been conducted to evaluate the financial burden that diabetes imposes on the Romanian National Health Insurance System, highlighting the significant challenges posed by this condition [4]. These data underscore that T2DM is a major health issue in Romania, necessitating the development of effective treatment strategies to mitigate its impact on both individuals and the national healthcare system. 

Managing T2DM goes beyond the mere intake of medication, considering that there is no known cure [5]. Developing healthy habits and maintaining psychological well-being are essential components for effectively managing diabetes [6]. This goal can only be reached with the help of medical nutrition therapy, regular exercise, counseling to help people quit smoking, psychological assistance, and support and guidance for diabetes self-management [7]. Studies have demonstrated the effectiveness of lifestyle interventions in controlling T2DM, with some cases even achieving complete remission through intensive lifestyle modifications [8]. Moreover, the impact of lifestyle-related factors extends to mortality outcomes in individuals with T2DM. Combined healthy lifestyle behaviors have been found to influence all-cause and cardiovascular-specific mortality in patients with T2DM, highlighting the importance of lifestyle choices in improving health outcomes [9].

Understanding local dietary patterns and behaviors can provide insights specific to the population, which is important for developing targeted interventions. In terms of diet, the key components of T2DM management and prevention are both eating behaviors and diet quality. Eating behavior plays a crucial role in maintaining health and preventing disease and is associated with obesity and other chronic diseases [10,11]. Various factors influence eating behaviors, which in turn shape eating habits, diet choices, and food-related decisions [12]. Regarding T2DM, an increased risk of disordered eating behaviors has been observed in individuals with diabetes; those who are already vulnerable to or experiencing disordered eating may find their condition worsened by diet and weight loss advice meant to manage the disease, regardless of how sincere or misinterpreted it may be [13].

A higher diet quality has been linked to a lower risk of T2DM [14], and better glucose control [15]. Furthermore, diet quality has been correlated with various metabolic biomarkers and risk factors in individuals with T2DM and low diet quality has been associated with increased odds of hyperglycemia, dyslipidemia, and excess weight in adults with T2DM [16]. Additionally, a high-quality diet is inversely related to the risk of cardiovascular disease and T2DM [17]. Various diet quality indices, such as the Healthy Eating Index (HEI) [18] and the Diet Quality Index-International (DQI-I) [19], have been developed to assess the dietary patterns of both healthy and unhealthy populations. Among these, the DQI-I offers a broader approach, as it captures different components of diet quality, including variety, adequacy, moderation, and balance [19]. These components are particularly significant in the context of the Romanian diet because they align with the key recommendations of the Romanian Dietary Guidelines for Healthy People [20]. The DQI-I provides a more comprehensive evaluation of the eating habits of a population, which can lead to more effective dietary interventions. Our previous research demonstrated a connection between eating behavior and dietary intake, but it primarily focused on the relationship between nutrients and food groups [21]. The DQI-I, by contrast, allows for a more nuanced analysis of dietary patterns, facilitating a deeper understanding of how eating behavior influences the management of T2DM.

Patients must adopt a high-quality diet to manage T2DM, which includes following healthy eating patterns and adhering to beneficial dietary behaviors. Adopting these habits can help regulate blood glucose levels and decrease the risk of complications related to type 2 diabetes. Understanding and improving eating behavior is a priority in public health and nutritional education strategies. Hence, the primary aim of our study was to provide a detailed and context-specific analysis of how diet quality and eating behavior interact among T2DM patients in Romania, and to examine the associations between these factors, and anthropometric and clinical factors, ultimately leading to more effective, culturally appropriate health interventions and policies tailored to the needs of the Romanian population.

## 2. Materials and Methods

### 2.1. Study Design

The study participants were individuals with T2DM, aged 18 years or older, who were recruited from the Clinical Centre for Diabetes, Nutrition, and Metabolic Diseases of St. Spiridon’s Hospital, Iasi, Romania. We included patients who had a prior diagnosis of T2DM and met diagnostic criteria defined by the World Health Organization (WHO) [22]. The study design, recruitment process, inclusion and exclusion criteria, and some methods of data collection have been reported previously [21]. Briefly, we informed and invited all patients who visited their diabetes specialist for periodic check-ups to participate. We excluded non-T2DM patients, pregnant or nursing patients, insulin-treated patients, patients with mental health issues, hearing disorders, eating behavior disorders, other pathologies that might have affected dietary intake, patients on a special diet, and patients with incomplete questionnaire responses. Patients who had previously been recruited were contacted by telephone to see if they would like to continue participating in this study by completing additional data and taking new measurements. A total of 314 individuals responded positively to our request, and a physical appointment was set up in the clinic. The Ethics Commission of the “Grigore T. Popa University of Medicine and Pharmacy” Iasi approved the research (82/20.05.2021), respecting all the requirements established in the Declaration of Helsinki. All patients signed informed consent before participating in this study.

### 2.2. Data Collection

#### 2.2.1. Sociodemographic Characteristics and Cardiovascular Risk Factors 

Age, sex, area of residence, employee status, smoking status, and alcohol consumption were declared by the participants before collecting other data. 

Anthropometric measurements were conducted using standardized procedures. Weight was measured with an electronic scale after the individuals removed their heavy clothes, shoes, and other personal objects to the nearest 0.1 kg accuracy. Height was measured with a stadiometer, to the nearest 0.1 cm, from the subject’s head to toe in an upright standing position, without shoes. Waist circumference (WC) was measured with a measuring tape at the end of a standard expiration, to the nearest 0.1 cm. The examiner sat in front of the subject while the WC was measured, placing the measuring tape horizontally, halfway between the iliac crest and the lowest rib margin. The body mass index (BMI) was calculated using the formula weight divided by height squared (kg/m^2^). Individuals were classified into two categories based on the cut-off value for obesity: without obesity, when BMI < 30 kg/m^2^, and with obesity, when BMI ≥ 30.0 kg/m^2^ [23]. 

The blood pressure (systolic blood pressure—SBP, and diastolic blood pressure—DBP) of the participants was measured using an automatic blood pressure monitor by a qualified nurse. The measurements were expressed in mmHg and taken after a minimum of 10 min of rest. The procedure consisted of collecting two measurements in the non-dominant arm and calculating the average of these two measurements.

The concentrations of fasting blood glucose (FBG), glycated hemoglobin (HbA_1_C), total cholesterol (TC), low-density cholesterol (LDL-c), high-density cholesterol (HDL-c), and triglyceride (TG), as well as the date of diagnosis were all retrieved from the health records of the Clinical Centre for Diabetes, Nutrition, and Metabolic Diseases of St. Spiridon’s Hospital, Iasi, Romania. The diabetes duration was calculated by subtracting the year of diagnosis from the year of data collection. The atherogenic index of plasma (AIP) was calculated using lipid profile data, according to the following formula: AIP = log(TG/HDL-c) in mg/dL [24]. AIP values below 0.11 indicated a low risk of cardiovascular disease (CVD); values between 0.11 and 0.21 indicated a moderate risk; and values above 0.21 indicated an increased risk of CVD. 

#### 2.2.2. Diet Characteristics

Dietary intake was assessed with EPIC Food Frequency Questionnaire (EPIC-FFQ), an FFQ with a 130-item food and beverages list [25]. The FFQ was previously adapted and validated in Romania, making it a reliable instrument for assessing dietary intake, including culturally specific dietary habits and consumption patterns [26]. Moreover, the EPIC-FFQ has been widely used in research on various chronic conditions, including diabetes. Its comprehensive ability to capture detailed information on food consumption and nutrient intake makes it an invaluable tool for assessing the nutritional status of individuals with T2DM [27,28]. A trained dietitian administered the FFQ face to face, explaining portion sizes and consumption frequency to all participants to minimize reporting errors. Participants picked a consumption frequency based on the previous year’s intake. FFQ Epic Tool for Analysis (FETA), version 6 [25] was used to calculate average daily intakes. Based on the data obtained with the FFQ and FETA, we used the DQI-I to determine the diet quality [19]. For the purpose of calculating DQI-I, the following nutrients and food groups were taken from the report generated by the FETA program: energy, proteins, total fats, saturated fatty acids (SFA), monounsaturated fatty acids (MUFA), polyunsaturated fatty acids (PUFA), cholesterol, fibers, iron, calcium, vitamin C, sodium, “Cereals and cereal products”, “Vegetables”, “Fruit”, “Meat and meat products”, “Fish and fish products”, “Eggs and eggs dishes”, “Milk and milk products”, “Fats and oils”, “Non-alcoholic beverages”, “Sugars; preserves and snacks”, “Alcoholic beverages”. In addition, information on poultry and beans intake was taken from the frequency reported by participants, as the FETA report does not generate daily consumption of this foods. Macronutrient intake was reported to total daily energy to determine the percentages of macronutrients in total energy. Food groups expressed in grams were converted to food portions given the FFQ associated portions. The following categories were considered in calculating the DQI-I total score: variety, adequacy, moderation, and overall balance. Variety has two subcomponents: food group variety and protein variety. Food group variety means that at least one serving of food per day from each of the five food groups (i.e., meat/poultry/fish/egg, dairy/beans, grains, fruits, and vegetables) is consumed and is scored with 3 points for each food group, totaling a subcomponent score between 0 and 15. Protein variety (i.e., meat, poultry, fish, dairy, beans, and eggs) refers to the intake of more than half the serving size per day from different protein sources; depending on the number of protein sources in the diet, a score between 0 and 5 is given, where the maximum score of 5 is given when at least 3 sources of protein are actually consumed. The total variety score is calculated by summing the food group variety score and the protein variety score. Adequacy includes eight components: fruit, vegetable, grain, and fiber intake, reported at three standard energy levels (1700/2200/2700 kcal), protein intake reported at daily energy intake, and iron, calcium, and vitamin C intake reported at the Recommended Daily Allowance (RDA), according to gender and age. We adjusted the food portions proportionately for individual energy levels for the following components: fruit, vegetable, grain, and fiber. For each component, a score between 0 and 5 points is awarded, and the total score for adequacy is calculated by summing the eight components. Moderation assesses the consumption of foods and nutrients that are linked to chronic diseases and may require limitation and it consists of five components: intake of total fat, cholesterol, saturated fat, sodium, and empty-calorie food. The highest intake for every component receives a score of 0, and the lowest intake category receives the highest score of 6. The cut-offs defined by Kim et al. were used [19], although the DQI-I uses stricter limits than other dietary indices. The maximum score is given when total fat accounts for 20% of total energy intake, and the lowest score is given when it reaches 30%. For saturated fat, the highest score is awarded if intake is ≤7% of total energy, and the lowest if >10%. For cholesterol and sodium, the highest score is given if intake is ≤300 mg of cholesterol or ≤2400 mg of sodium daily. “Empty-calorie foods” are those with poor nutrient density, providing energy without necessary nutrients, and the score was calculated from the intake of sugar, sweets, oils, and alcohol. The highest score of 6 is awarded if the energy provided by these foods makes up ≤3% of total daily energy intake, and the lowest score of 0 if it exceeds 10%. All moderation scores are summed up for the total moderation score, which ranges between 0 and 30 points. Overall balance has two components: the macronutrient ratio, with a score between 0 and 6 points, and fatty-acid ratio, with a score between 0 and 4 points. The macronutrient ratio is based on the proportions of energy derived from carbohydrates, proteins, and fats. A score of 6 is awarded when the diet consists of 55–65% carbohydrates, 10–15% proteins, and 15–25% fats. The fatty-acid ratio is based on the proportions of fatty acids (i.e., PUFA, MUFA, and SFA). When the PUFA/SFA ratio and the MUFA/SFA ratio both fall between 1 and 1.5, the maximum score of 4 is given. A total score for overall balance is derived from these two components. The 4 components scores (i.e., variety, adequacy, moderation, and overall balance) were summed up to obtain the total DQI-I score. A score between 0 and 100 points was obtained for each individual, with higher scores indicating better overall diet quality. 

The Dutch Eating Behavior Questionnaire (DEBQ), previously validated [29,30], was used for the assessment of eating behavior. The questionnaire contains 33 items distributed among three main scales: “Emotional eating” (EmoE) measures excessive food consumption as a result of emotional factors; “External eating” (ExtE) measures overeating in response to food-related cues; “Restrained eating” (RE) measures attempts to control overeating. Each item is assigned a value from 1 to 5 (never–very often) on a five-point Likert scale. Higher scores indicate a higher level of eating style (i.e., emotional, external, or restrained eating).

#### 2.2.3. Physical Activity Level

The level of physical activity was assessed with the International Physical Activity Questionnaire Long Form (IPAQ-L) [31,32]. The IPAQ-L questionnaire includes 27 questions that assess the level of physical activity undertaken in the past week. The sub-scores in MET-minutes per week were determined by multiplying the Metabolic Equivalent of Task (MET) values, which were provided in the scoring protocol (i.e., walking = 3.3, moderate activity = 4, vigorous activity = 8), by the duration of the activity in minutes, and the number of days it was performed. Sub-scores were calculated for walking, moderate-intensity activity, vigorous-intensity activity, and each domain (work, transportation, leisure time, domestic, and gardening). The total score was calculated by adding up the MET minutes of physical activity per week for walking, moderate activity, and vigorous activity. 

### 2.3. Statistical Analysis

The statistical analysis was conducted using version 20 of the SPSS (Statistical Package for the Social Sciences) software (IBM, Armonk, N.Y., USA). The mean ± standard deviation (SD) was used to express continuous variables, while numbers and proportions were used for categorical variables. The participant’s characteristics were compared using Chi-squared and *t*-tests. The Levene test was employed to assess the homogeneity of variances, and the normality of the variable distribution was confirmed through the Kolmogorov–Smirnov test. A one-way ANOVA was employed to compare the differences in means between three or more groups. After the ANOVA and based on the homogeneity of variances, we used post hoc tests to find the specific differences between the groups: Tukey if Levene’s test showed that the variances were equal (*p* > 0.05) or the Games–Howell test if Levene’s test indicated that the variances were unequal (*p* ≤ 0.05). Spearman correlations were calculated to assess the associations among diet quality, eating behavior, and cardiovascular risk factors. Additionally, we used a multiple linear regression model to test the relationship between diet quality and eating behavior in three models: a crude model and two adjusted models. In the crude model, diet quality components were used as dependent variables, and eating behavior components were used as predictors. The following covariates were included in the adjusted models: age, area of residence, duration of diabetes, employee status, daily energy intake, and physical activity level (Model 1); plus sex (Model 2). Statistical hypotheses were tested using a confidence interval of 95%, and *p*-values < 0.05 were considered statistically significant.

## 3. Results 

### 3.1. Socio-Demographic, Anthropometric, and Biochemical Characteristics of the Study Population

Table 1 presents the descriptive characteristics of the study population. Of the 314 patients with T2DM, 43.6% (*n* = 137) were men, and 56.4% (*n* = 177) were women. The average duration of diabetes was 7.45 years. The mean age was 61.89 years, with women having a higher age than men (62.91 vs. 60.58, *p* = 0.035). Women had a significantly higher TC (200.37 vs. 187.82, *p* = 0.019) and HDL-c (51.59 vs. 45.51, *p* < 0.001). There were no statistically significant differences between men and women regarding diabetes control (*p* > 0.05).

### 3.2. Diet Quality of the Study Population

A one-sample *t*-test was performed to compare the total DQI-I score with the maximum score. The mean score of Total DQI-I was 60.21, and it was significantly lower than the maximum possible score: *t* (313) = −91.29, *p* < 0.001. The component protein adequacy did not have any variation in the studied population, so it was excluded from further analysis. Women had a significantly higher diet quality than men (61.40 vs. 58.68, *p* = 0.002). For DQI-I components, women also had a significantly higher score for vitamin C adequacy (4.32 vs. 3.70, *p* < 0.001), total moderation (14.00 vs. 10.77, *p* < 0.001), total fat moderation (1.51 vs. 1.12, *p* = 0.032), cholesterol moderation (4.58 vs. 3.50, *p* < 0.001), sodium moderation (3.71 vs. 2.56, *p* < 0.001) and macronutrient ratio (0.20 vs. 0.04, *p* = 0.020) (Table 2).

Patients from rural areas had a significantly higher score for adequacy when compared with patients from urban areas (28.60 vs. 28.55, *p* = 0.048). Also, patients who were retired had a higher score for moderation when compared with employees (13.15 vs. 10.86, *p* = 0.022). Depending on alcohol consumption, we found that patients who do not consume alcohol had a better (61.81 vs. 58.51, *p* < 0.001) and more moderate (14.50 vs. 10.56, *p* < 0.001) diet quality compared to those who consume alcohol (Table 3).

### 3.3. Eating Behavior of the Study Population

In the total study population, mean scores for eating behavior were 1.53 ± 0.81 for emotional eating, 2.38 ± 1.00 for external eating, and 2.24 ± 0.96 for restrained eating. Women had a significantly higher score for emotional eating (2.00 vs. 1.53, *p* < 0.001) and restrained eating (2.39 vs. 2.05, *p* = 0.002). Patients from urban areas had significantly higher scores for emotional eating (1.89 vs. 1.63, *p* = 0.034). After splitting the population into people with and without obesity, we found that people with obesity had a significantly higher score for restrained eating compared with patients without obesity (2.38 vs. 2.04, *p* = 0.002) (Table 4).

### 3.4. Associations between Eating Behavior and Anthropometric and Biochemical Data

**Figure 1:**
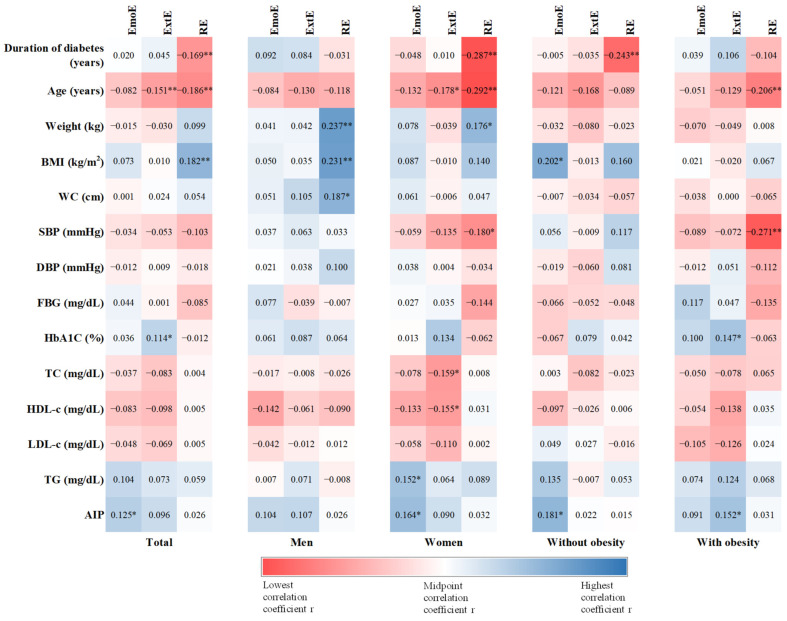
Heat map of Spearman correlation between eating behavior and anthropometric and biochemical data. Correlation coefficient r is presented in the square and the strength of correlation is depicted in color, with red being the lowest and blue the highest correlation coefficient. Statistical significance: * *p* < 0.05, ** *p* < 0.01. EmoE—Emotional eating, ExtE—External Eating, RE—Restrained Eating, BMI—body mass index, WC—waist circumference, SBP—systolic blood pressure, DBP—diastolic blood pressure, FBG—fasting blood glucose, HbA_1_C—glycated hemoglobin, TC—total cholesterol, LDL-c—low-density cholesterol, HDL-c—high-density cholesterol, TG—triglyceride, AIP—atherogenic index of plasma.

Duration of diabetes correlated negatively with restrained eating in the total study population (r = −0.169, *p* = 0.003), in the women group (r = −0.287, *p* < 0.001) and also for patients without obesity (r = −0.243, *p* = 0.006). For age, we found that people with external and restrained eating have a lower age (ExtE: r = −0.151, *p* = 0.007; RE: r = −0.186, *p* = 0.001). Moreover, age was negatively correlated with external eating (r = −0.178, *p* = 0.018) and restrained eating (r = −0.292, *p* < 0.001) in women, and only with restrained eating in patients with obesity (r = −0.206, *p* = 0.005). Weight was positively correlated with restrained eating in men (r = 0.237, *p* = 0.005) and in women (r = 0.176, *p* = 0.019) and BMI was also positively correlated with restrained eating in the total group (r = 0.182, *p* = 0.001) and in men (r = 0.231, *p* = 0.007), and with emotional eating in patients without obesity (r = 0.202, *p* = 0.022). Men with higher WC have a higher score for restrained eating (r = 0.187, *p* = 0.033). SBP negatively correlated with restrained eating in women (r = −0.180, *p* = 0.017) and patients with obesity (r = −0.271, *p* < 0.001). HbA_1_C positively correlated with external eating in the total group (r = 0.114, *p* = 0.044) and in patients with obesity (r = 0.147, *p* = 0.045). We found that women with higher external eating had a lower TC (−0.159, *p* = 0.034) and HDL-c (r = −0.155, *p* = 0.030), and women with higher emotional eating had a higher TG level (r = 0.152, *p* = 0.043). AIP positively correlated with emotional eating in the total group (r = 0.125, *p* = 0.030), in women (r = 0.164, *p* = 0.032), and in patients without obesity (r = 0.181, *p* = 0.045). Also, AIP positively correlated with external eating, but just for patients with obesity (r = 0.152, *p* = 0.042) (Figure 1). 

### 3.5. Associations between Eating Behavior and Diet Quality

The correlations between eating behavior and diet quality, along with the results of the linear regression from the unadjusted model (crude model), are presented in the Supplementary Materials (Figure S1, Table S1). Significant associations and relationships were found between various eating behaviors and diet quality components: emotional eating with total adequacy, grain, fiber, and vitamin C adequacy, empty-calorie foods moderation; external eating with total adequacy, grain, fiber, and calcium adequacy, total moderation, sodium and empty-calorie moderation, fatty-acid ratio and overall balance score; restrained eating with vegetable, vitamin C, and calcium adequacy. 

Table 5 presents the results of the linear regression between diet quality components and eating behavior for Model 1, adjusted for covariates (i.e., age, duration of diabetes, area of residence, employee status, daily energy intake, and physical activity level). Emotional eating was positively associated with vitamin C adequacy (β = 0.117, 95%CI: 0.003–0.289, *p* = 0.045) and sodium moderation (β = 0.103, 95%CI: 0.026–0.395, *p* = 0.026). We found a positive association between external eating and grain adequacy (β = 0.208, 95%CI: 0.126–0.403, *p* < 0.001) and negative associations with empty-calorie food moderation (β = −0.125, 95%CI: −0.474–−0.022, *p* = 0.032) and fatty-acid ratio (β = −0.151, 95%CI: −0.305–0.042, *p* = 0.010). Regarding the association between restrained eating and diet quality, we found one positive association with vitamin C adequacy (β = 0.138, 95%CI: 0.034–0.346, *p* = 0.017), and also with fruit adequacy (β = 0.125, 95%CI: 0.013–0.306, *p* = 0.033).

In Model 2, after adding sex as a covariable, only the relationships with external eating were kept. For grain adequacy, empty-calorie food, and fatty-acid ratio, the results were similar with the one from Model 1. Additionally, a relationship between external eating and food group variety was found (β = 0.185, 95%CI: −0.404–−0.001, *p* = 0.049). The results of Model 2 are presented in the Supplementary Materials (Table S2).

## 4. Discussion

In this cross-sectional study, we explored the relationship between diet quality and eating behavior in people with T2DM. A deeper analysis showed that emotional, external, and restrained eating are linked to several diet quality components. DQI-I helps determine if a person’s diet meets nutritional guidelines. In our study, the participants’ mean diet quality score was significantly lower than the maximum achievable score, indicating a reduced diet quality. Our study found no variation in the component protein adequacy, a result that could reflect cultural differences in protein intake among our population. On that note, according to Petrescu et al., meat is deeply rooted in Romanian cuisine and used in various traditional dishes [33].

The gender differences identified in our study showed that women have a higher diet quality, better fruit and vegetable intake, and better moderation for fat and sodium compared to men. Our results support prior studies indicating that women have a better diet quality than men, especially in terms of fruit and vegetables [34], moderation, and fats from meat [35,36]. This may be attributed to the fact that women tend to be more health-conscious than men [37]. Men’s higher iron and calcium adequacy scores can be attributed to their inclination towards consuming animal-derived foods [38,39]. 

We also found differences in diet quality from a socioeconomic perspective. Rural patients scored significantly higher for adequacy than urban patients. Research suggests that rural communities can benefit from direct access to healthier agricultural products, which may increase nutritional intake [40]. Other research showed that rural diets were better than urban diets [41], and that local food culture from rural diets has an important impact on healthy eating habits [42]. Additionally, we also found that retired patients scored better on moderation than employees. The more time retired people have to prepare meals and their potential to prioritize food quality may explain this outcome. In contrast, employees may choose fast and unhealthy options due to time constraints [43]. In relation to alcohol consumption, we found that non-drinkers exhibited superior overall diet quality and better moderation compared to drinkers. These results align with the prevailing research showing that alcohol-free people live healthier [44]. 

Our study found that women had higher emotional and restrained eating. Studies suggest that emotional eating is more common in women than men, probably due to socio-cultural and hormonal differences [45]. Patients in urban areas showed higher scores for emotional eating. Studies suggest that urbanization impacts eating habits [46,47]. Our study found that individuals with obesity exhibited higher restrained eating than non-obese individuals. This suggests that people with obesity may try to restrict food intake, which may lead to yo-yo dieting and weight fluctuations [48,49,50].

Our correlations analysis shows how diabetes duration and metabolic parameters affect eating behavior. Thus, we find that duration of diabetes was negatively correlated with restricted eating in the study population, in women and non-obese patients. Long-term diabetes management may reduce the need to restrict diet, possibly due to better disease management strategies [51,52]. In our study, higher HbA1C levels were associated with more external eating. A recent study found that mindful eating can improve glycemic control in T2DM patients [53]. We also found associations between external and emotional eating and lipid profile of the patients. Research led by Mensorio et al. [54] indicated that emotional eating can increase total and LDL cholesterol. We found a positive correlation between weight and BMI and restrained eating, suggesting that individuals with a higher body weight may be more likely to attempt weight control through dietary restriction. Snoek et al. argue that a higher BMI predicts more restrained eating [55] and this can lead to cycles of restriction and overeating [56], which hinder long-term weight management [57]. Our results show that men with a larger waist circumference tend to engage in restricted eating behaviors more often, which may indicate attempts to control weight or manage food intake more strictly. Restrained eating was negatively correlated with systolic blood pressure, in women and patients with obesity. These results can be explained by the fact that adopting a more restrictive diet can lead to lower blood pressure values [58], a known risk factor for many chronic diseases [59]. Our results indicate that the AIP positively correlated with emotional eating in the total study group, in women, and in non-obese patients, and with external eating, only in obese patients. This suggests a higher risk of cardiovascular disease for emotional and external eaters and could reflect a tendency for those patients to choose unhealthy foods, which contributes to increased atherogenic risk. Wu et al. showed that emotional eating is associated with cardiovascular risk factors [60]. Moreover, individuals with emotional and external eating are more likely to eat ultra-processed foods, foods that could enhanced cardiovascular disease risk [61]. Therefore, the current findings emphasize the need for an integrated and personalized approach to diabetes management, which includes psychological support.

In our study, age was negatively correlated with external eating, and restrained eating, especially in women, indicating that younger people may be more susceptible to external eating influences. Studies have indicated that emotional eating decreases with age [62], and older people eat less externally than younger people [63]. This may reflect greater social and emotional pressures on young people.

The correlations and regression analysis results between eating behavior and diet quality components initially revealed important relationships. However, after adjusting for covariates, many of these initial associations lost significance. We found that patients with higher emotional eating scores had higher vitamin C adequacy scores. Our results are in contrast to prior research indicating that emotional eaters typically consume lower amounts of vitamin C and other nutrients [64]. This highlights a possible role for vitamin C consumption in managing emotion through food, possibly due to vitamin C’s antioxidant effects [65]. Emotional eating might increase fruit and citrus consumption, explaining higher vitamin C adequacy scores. Emotional eating is often characterized by a high consumption of hyperpalatable, caloric foods in response to negative emotions [66] and individuals may opt for foods low in essential nutrients, without considering their impact on health [67]. However, other studies indicate that emotional eating can be associated with the presence of positive emotions [68]. Mastinu et al. linked scent memories of specific fruits, vegetables, and pastries to positive emotions [69]. A similar result was observed by Nguyen et al. in boys with emotional eating [70]. These mixed results show the complexity of the eating behavior, and the need to better understand the reasons why people resort to emotional eating.

In terms of external eating, these patients were found to have higher grain adequacy scores. After adding sex as a covariate, external eating was positively associated with food group variety. On the other hand, external eating negatively correlated with empty-calorie food moderation and fatty-acid ratio. These results suggest that people who eat in response to external stimuli tend to consume grains regularly. Our study’s results may be influenced by how grains are categorized in the dietary analysis, grouping refined and whole grains together. Whole grains are generally associated with greater health benefits, while refined grains may have less beneficial effects [71]. The negative associations with empty-calorie foods and fatty-acid ratio indicate a tendency for people with external eating to consume less healthy foods. External eating has been associated with increased consumption of unhealthy foods [72,73]. A recent study revealed that individuals with greater disinhibition and hunger levels exhibited a lower dietary quality [74]. Moreover, Paans et al. linked emotional and external eating to snacking and fast-food consumption [75]. Due to their heightened sensitivity to external cues, those with external eating may have a more varied diet. The findings of our study are in line with other research that emphasizes the complexity of eating behavior and highlights the need for a different approach in nutritional counseling [76,77]. 

For patients with restrained eating, we found positive relationships with fruit and vitamin C adequacy. Yong et al. found comparable findings, specifically that students exhibiting high levels of restrained eating displayed a greater tendency to consume fruits on a more frequent basis [56]. Restrained eaters may be more aware of the quality of the foods they eat, avoiding those with low nutritional value [78]. Individuals with high levels of restrained eating are more likely to have improved diet quality, while those with low levels of restrained eating tend to have poor diet quality due to a lack of conscious control over eating [79]. This suggests that people with restrained eating make a conscious effort to include fruits and sources rich in vitamin C in their diet in an attempt to maintain a balanced diet while at the same time limiting other types of food.

### Strengths and Limitations

The current study offers several significant strengths. This is the first study to look into the link between eating behaviors, diet quality, and cardiovascular risk factors in Romanian T2DM, according to the authors’ knowledge. We adjusted the analysis for various confounding factors that could influence diabetes control, such as exercise, smoking, and alcohol consumption, to minimize bias and ensure the reliability of the results. Furthermore, we used validated qualitative tools that were appropriate for the study population and allowed for a comprehensive understanding of the health and nutritional status of patients with T2DM. Nevertheless, our study has several limitations. This study’s cross-sectional nature and single-time data collection limit its ability to establish causation. Furthermore, questionnaires may introduce biases that reduce the accuracy of findings. FFQs depend on participants’ capacity to remember their food consumption, using a pre-established inventory of food items and drinks. This non-exhaustive list may not include all eaten foods. Therefore, the DQI-I derived from an FFQ may not precisely reflect the actual dietary habits. Nevertheless, FFQs may lack comprehensive data regarding the nutrient density or the difference in the quality of consumed foods, and also do not account for added salt or other seasonings, or the use of dietary supplements. Despite our attempts to account for confounding factors in the analysis, we cannot rule out the possibility of unmeasured or residual confounding variables influencing the observed relationships in this study. Furthermore, our study missed the inclusion of essential features (i.e., education, nutritional knowledge, and stress) that could potentially influence our findings. Another limitation of our study is the lack of similar research; without comparative studies, it is challenging to contextualize findings and draw robust conclusions. Also, our study primarily focused on characteristics related to dietary habits, neglecting to assess other potentially significant factors like adherence to medical treatment. Finally, participants from a single healthcare center restrict the applicability of the findings to a wider population of Romanian patients with T2DM. 

The results of our study responded to the main aim and emphasize the need for a more personalized approach to nutritional counseling that takes into account the impact of eating behavior on diet quality. Attention must be focused on improving moderation of less healthy foods, especially for external eaters. This is crucial for more efficient metabolic management and overall health. 

## 5. Conclusions

Our study explored the associations between eating behaviors and diet quality among individuals with T2DM in Romania. To our knowledge, it is the first study to investigate these associations using validated tools—DEBQ, EPIC FFQ, and a reliable diet quality score, DQI-I. The analysis of this index, as well as the eating behavior of patients with T2DM, can provide an image of the current nutritional status but can also guide interventions to improve eating habits, significantly contributing to diabetes control and improving the quality of life. Our results underscore the complex interactions between different eating behaviors and diet quality components, highlighting the need for personalized dietary interventions that consider individual eating patterns to improve overall nutritional status and health outcomes for patients with T2DM. 

## Figures and Tables

**Table 1:** Descriptive characteristics of the study population (*n* = 314).

		Total	Men	Women	*p*-Value
Duration of diabetes (years)		7.45 ± 5.70	7.20 ± 5.29	7.64 ± 6.01	0.490
Age (years)		61.89 ± 9.69	60.58 ± 10.61	62.91 ± 8.81	**0.035**
Duration of diabetes		7.45 ± 5.70	7.20 ± 5.28	7.64 ± 6.01	0.497
Weight (kg)		87.34 ± 18.25	95.20 ± 18.42	81.25 ± 15.64	**<0.001**
BMI (kg/m^2^)		31.79 ± 5.52	31.45 ± 5.43	32.05 ± 5.59	0.343
WC (cm)		107.27 ± 14.12	111.26 ± 12.88	104.23 ± 14.29	**<0.001**
SBP (mmHg)		141.06 ± 20.34	142.49 ± 21.68	139.99 ± 19.27	0.287
DBP (mmHg)		83.71 ± 12.38	86.71 ± 12.12	81.47 ± 12.13	**<0.001**
FBG		149.31 ± 46.47	149.15 ± 53.08	149.44 ± 40.78	0.956
HbA_1_C		7.29 ± 1.58	7.29 ± 1.71	7.29 ± 1.49	0.977
TC (mg/dL)		194.89 ± 47.09	187.82 ± 38.59	200.37 ± 52.18	**0.019**
HDL-c (mg/dL)		48.93 ± 14.26	45.51 ± 12.53	51.59 ± 14.97	**<0.001**
LDL-c (mg/dL)		120.28 ± 39.69	118.89 ± 34.70	121.35 ± 43.20	0.592
TG (mg/dL)		165.99 ± 87.35	156.72 ± 73.76	173.16 ± 96.16	0.098
AIP		0.49 ± 0.27	0.50 ± 0.26	0.49 ± 0.28	0.652
Daily energy intake (kcal)		1581.71 ± 619.83	1678.71 ± 500.94	1506.62 ± 690.18	**0.014**
Physical activity level (MET)		3369.68 ± 3351.62	4235.99 ± 3970.90	2699.15 ± 2599.38	**<0.001**
Area of residence	Urban	64.00 (201)	67.9 (93)	61.0 (108)	0.209
	Rural	36 (113)	32.1 (44)	39.0 (69)	
Employee-status	Employee	25.2 (79)	38.7 (53)	14.7 (26)	**<0.001**
	Retired	65.6 (206)	54.7 (75)	74.0 (131)	
	Unemployed	9.2 (29)	6.6 (9)	11.3 (20)	
Smoking status	Non-smoker	90.4 (284)	84.7 (116)	94.9 (168)	**0.005**
	Smoker	7.6 (24)	13.1 (18)	3.4 (6)	
	Undeclared	1.9 (6)	2.2 (3)	1.7 (3)	
Alcohol consumption	No	51.6 (162)	27.00 (37)	70.6 (125)	**<0.001**
	Yes	48.4 (152)	73.00 (100)	29.4 (52)	
BMI status	Without obesity	40.8 (128)	43.8 (60)	38.4 (68)	0.356
	With obesity	59.2 (186)	56.2 (77)	61.6 (109)	

Continuous variables are presented as mean ± SD, and categorical data as percentages (%) and numbers (*n*). BMI—body mass index, WC—waist circumference, SBP—systolic blood pressure, DBP—diastolic blood pressure, FBG—fasting blood glucose, HbA_1_C—glycated hemoglobin, TC—total cholesterol, LDL-c—low-density cholesterol, HDL-c—high-density cholesterol, TG—triglyceride, AIP—atherogenic index of plasma. The means of continuous variables were compared using the Independent *t*-test and categorical variables were compared using the Chi-square test. Bold values denote statistical significance at the *p* < 0.05 level.

**Table 2:** Diet quality of the study population (*n* = 314).

Component	Score	Total	Men	Women	*p*-Value
Variety	0–20	18.31 ± 2.29	18.28 ± 2.36	18.33 ± 2.24	0.831
Food groups variety	0–15	13.71 ± 1.72	13.64 ± 1.81	13.76 ± 1.64	0.540
Protein sources variety	0–5	4.60 ± 0.98	4.64 ± 0.91	4.57 ± 1.04	0.567
Adequacy	0–40	28.57 ± 4.94	28.84 ± 4.98	28.36 ± 4.92	0.392
Vegetable group	0–5	4.33 ± 1.12	4.15 ± 1.19	4.46 ± 1.06	**0.017**
Fruit group	0–5	4.45 ± 1.23	4.28 ± 1.38	4.58 ± 1.08	**0.035**
Grain group	0–5	0.67 ± 1.27	0.80 ± 1.36	0.57 ± 1.20	0.116
Fiber	0–5	3.56 ± 1.19	3.64 ± 1.09	3.51 ± 1.27	0.354
Protein	0–5	5.00 ± 0.00	5.00 ± 0.00	5.00 ± 0.00	-
Iron	0–5	4.45 ± 1.02	4.78 ± 0.62	4.19 ± 1.18	**<0.001**
Calcium	0–5	2.05 ± 1.58	2.49 ± 1.49	1.72 ± 1.58	**<0.001**
Vitamin C	0–5	4.05 ±1.32	3.70 ± 1.42	4.32 ± 1.18	**<0.001**
Moderation	0–30	12.59 ± 6.54	10.77 ± 6.84	14.00 ± 5.94	**<0.001**
Total fat	0–6	1.34 ± 1.60	1.12 ± 1.58	1.51 ± 1.60	**0.032**
Saturated fat	0–6	1.67 ± 1.91	1.45 ± 1.92	1.85 ± 1.89	0.065
Cholesterol	0–6	4.11 ± 2.23	3.50 ± 2.25	4.58 ± 2.09	**<0.001**
Sodium	0–6	3.21 ± 2.16	2.56 ± 2.13	3.71 ± 2.04	**<** **0.001**
Empty-calorie foods	0–6	2.26 ± 1.99	2.15 ± 1.99	2.36 ± 1.99	0.355
Overall balance	0–10	0.75 ± 1.31	0.79 ± 1.35	0.71 ± 1.28	0.613
Macronutrient ratio	0–6	0.13 ± 0.65	0.04 ± 0.38	0.20 ± 0.80	**0.020**
Fatty-acid ratio	0–4	0.61 ± 1.14	0.74 ± 1.23	0.51 ± 1.06	0.076
Total DQI-I score	0–100	60.21 ± 7.72	58.68 ± 8.24	61.40 ± 7.09	**0.002**

Data are presented as mean ± SD. SD—standard deviation, DQI-I—Diet Quality Index-International. The means of the variables were compared using the Independent *t*-test. Bold values denote statistical significance at the *p* < 0.05 level.

**Table 3:** Comparison of diet quality component scores.

Variables		Total DQI	Variety	Adequacy	Moderation	Overall Diet
Area of residence	Urban	60.31 ± 7.96	18.37 ± 2.32	28.55 ± 5.06 ^a^	2.61 ± 6.52	0.78 ± 1.32
	Rural	60.04 ± 7.29	18.19 ± 2.25	28.60 ± 4.75 ^b^	12.56 ± 6.60	0.69 ± 1.30
Employee status	Employee	58.87 ± 7.36	18.15 ± 2.36	29.00 ± 4.96	10.86 ± 6.10 ^a^	0.86 ± 1.38
	Retired	60.63 ± 7.81	18.31 ± 2.33	28.50 ± 4.90	13.15 ± 6.59 ^b^	0.67 ± 1.20
	Unemployed	60.93 ± 7.82	18.76 ± 1.82	18.76 ± 1.82	13.34 ± 6.68	0.97 ± 1.82
Smoking status	Smoker	60.25 ± 7.62	18.29 ± 2.30	28.47 ± 4.96	12.76 ± 6.42	0.73 ± 1.25
	Non-smoker	59.54 ± 7.91	18.29 ± 2.27	30.42 ± 3.94	10.00 ± 7.48	0.83 ± 1.85
	Undeclared	61.00 ± 12.37	19.17 ± 2.04	25.83 ± 6.24	15.00 ± 6.57	1.00 ± 1.67
Alcohol consumption	No	61.81 ± 7.67 ^a^	18.34 ± 2.22	28.12 ± 4.63	14.50 ± 5.99 ^a^	0.85 ± 1.33
	Yes	58.51 ± 7.42 ^b^	18.28 ± 2.38	29.04 ± 5.24	10.56 ± 6.50 ^b^	0.63 ± 1.29
BMI Status	Without obesity	60.52 ± 8.09	18.25 ± 2.31	28.58 ± 4.98	12.98 ± 6.47	0.70 ± 1.11
	With obesity	60.01 ± 7.47	18.35 ± 2.27	28.56 ± 4.93	12.32 ± 6.58	0.77 ± 1.44

Data are presented as mean ± SD. SD—standard deviation, BMI—body mass index. The means of continuous variables were compared using the Independent *t*-test and One-Way ANOVA (post hoc comparisons were conducted using the Tukey test when equal variances were assumed and the Games–Howell test when equal variances were not assumed). ^a, b^ indicates significant difference between groups.

**Table 4:** Eating behavior scores in the study population.

	Gender			Area of Residence			BMI Status		
	Men	Women	*p*-Value	Urban	Rural	*p*-Value	Without Obesity	With Obesity	*p*-Value
EmoE	1.53 ± 0.81	2.00 ± 1.18	**<0.001**	1.89 ± 1.04	1.63 ± 1.06	**0.034**	1.72 ± 1.00	1.85 ± 1.10	0.277
ExtE	2.30 ± 0.96	2.45 ± 1.02	0.175	2.44 ± 1.00	2.28 ± 0.98	0.179	2.36 ± 1.03	2.40 ± 0.98	0.734
RE	2.05 ± 0.89	2.39 ± 0.99	**0.002**	2.29 ± 0.94	2.15 ± 0.99	0.228	2.04 ± 0.85	2.38 ± 1.01	**0.002**

Data are presented as mean ± SD. SD—standard deviation, EmoE—Emotional eating, ExtE—External Eating, RE—Restrained Eating. The means of the variables were compared using the Independent *t*-test. Bold values denote statistical significance at the *p* < 0.05 level.

**Table 5:** Regression analysis of the associations between eating behavior and diet quality for Model 1.

	EmoE				ExtE				RE			
DQI-I Component	β	95%CI	Partial R^2^	*p*-Value	β	95%CI	Partial R^2^	*p*-Value	β	95%CI	Partial R^2^	*p*-Value
Variety	−0.004	−0.262–0.243	0.016	0.942	−0.095	−0.485–0.051	0.024	0.112	−0.015	−0.312–0.240	0.016	0.800
Food groups	−0.013	−0.210–0.168	0.018	0.825	−0.111	−0.391–0.009	0.029	0.061	−0.032	−0.264–0.149	0.019	0.586
Protein sources	0.013	−0.097–0.121	0.010	0.829	−0.026	−0.142–0.090	0.010	0.664	0.021	−0.098–0.141	0.010	0.722
Adequacy	0.040	−0.306–0.683	0.186	0.454	0.092	−0.070–0.980	0.192	0.089	0.055	−0.258–0.823	0.187	0.305
Vegetable group	0.056	−0.064–0.183	0.026	0.342	0.084	−0.037–0.226	0.030	0.157	0.075	−0.047–0.223	0.028	0.202
Fruit group	0.026	−0.105–0.164	0.027	0.665	−0.026	−0.176–0.111	0.027	0.655	0.125	0.013–0.306	0.041	**0.033**
Grain group	0.090	−0.024–0.241	0.128	0.107	0.208	0.126–0.403	0.159	**<0.001**	−0.040	−0.198–0.093	0.122	0.476
Fiber	0.051	−0.064–0.179	0.160	0.353	0.087	−0.026–0.233	0.165	0.116	0.081	−0.032–0.234	0.164	0.136
Iron	−0.078	−0.178–0.028	0.175	0.152	0.002	−0.108–0.111	0.169	0.977	−0.044	−0.159–0.066	0.171	0.414
Calcium	−0.093	−0.288–0.010	0.284	0.068	−0.041	−0.224–0.095	0.278	0.425	−0.095	−0.319–0.007	0.285	0.060
Vitamin C	0.117	0.003–0.289	0.055	**0.045**	0.066	−0.065–0.241	0.047	0.259	0.138	0.034–0.346	0.060	**0.017**
Moderation	0.059	−0.195–0.925	0.403	0.200	−0.036	−0.832–0.363	0.401	0.440	0.048	−0.288–0.938	0.402	0.298
Total fat	−0.015	−0.192–0.147	0.093	0.797	0.023	−0.143–0.217	0.093	0.686	−0.048	−0.264–0.106	0.095	0.401
Saturated fat	0.082	−0.050–0.347	0.126	0.141	0.017	−0.179–0.245	0.120	0.759	−0.007	−0.231–0.204	0.120	0.902
Cholesterol	0.087	−0.018–0.383	0.342	0.074	0.019	−0.171–0.258	0.336	0.692	0.059	−0.084–0.356	0.339	0.225
Sodium	0.103	0.026–0.395	0.404	**0.026**	−0.046	−0.298–0.098	0.396	0.321	0.058	−0.073–0.334	0.398	0.209
Empty-calorie foods	−0.082	−0.368–0.059	0.068	0.155	−0.125	−0.474–−0.022	0.076	**0.032**	0.073	−0.082–0.385	0.067	0.202
Overall balance	0.033	−0.103–0.185	0.020	0.577	−0.086	−0.267–0.040	0.026	0.146	0.012	−0.142–0.174	0.019	0.843
Macronutrient ratio	0.053	−0.039–0.105	0.017	0.374	0.091	−0.017–0.136	0.022	0.127	−0.020	−0.092–0.065	0.015	0.739
Fatty-acid ratio	0.008	−0.116–0.133	0.033	0.895	−0.151	−0.305–0.042	0.054	**0.010**	0.025	−0.107–0.166	0.034	0.673
Total DQI-I score	0.080	−0.228–1.399	0.096	0.158	−0.014	−0.979–0.760	0.091	0.804	0.073	−0.303–1.478	0.095	0.195

DQI-I—diet quality index-international, β—standardized coefficients, CI—confidence interval, R2—coefficient of determination, EmoE—Emotional eating. DQI-I—Diet quality index-international. Linear regression model and enter method were used. Model 1—adjusted for age, duration of diabetes, area of residence, employee status, daily energy intake, and physical activity level. Bold values denote statistical significance at the *p* < 0.05 level.

## Data Availability

Data are contained within the article and Supplementary Materials.

## Supplementary Materials

The following supporting information can be downloaded at: https://www.mdpi.com/article/10.3390/nu16132047/s1, Figure S1: Heat map of correlation between eating behavior and diet quality. Correlation coefficient r is presented in the square and the strength of correlation is depicted in color, with red being the lowest and blue the highest correlation coefficient: * *p* < 0.05, ** *p* < 0.01. EmoE—Emotional eating, ExtE—External Eating, RE—Restrained Eating, DQI-I—Diet Quality Index–International; Table S1: Regression analysis of the associations between eating behavior and diet quality for the crude model; Table S2: Regression analysis of the associations between eating behavior and diet quality for Model 2.
