# Supplementary material for: Association between Diet Quality and Eating Behavior in Type 2 Diabetes Adults: A Cross-Sectional Study
Source: Nutrients. 2024 Jun 27;16(13):2047. doi: 10.3390/nu16132047 (PMC11243329; doi:10.3390/nu16132047)
Supplement: Supplementary file 1 [file nutrients-16-02047-s001.zip › nutrients-3038450-supplementary.pdf]

	Total			Men			Women			Without obesity			With obesity		
	EmoE	ExtE	RE	EmoE	ExtE	RE	EmoE	ExtE	RE	EmoE	ExtE	RE	EmoE	ExtE	RE
Food groups variety	−0.042	−0.111	−0.057	−0.218*	−0.205*	−0.061	0.065	−0.042	−0.072	−0.006	−0.131	−0.035	−0.070	−0.093	−0.093
Protein sources variety	−0.002	−0.010	0.004	−0.169*	−0.155	−0.037	0.102	0.094	0.039	−0.078	0.003	0.055	0.043	−0.013	−0.023
Total variety	−0.033	−0.090	−0.044	−0.226**	−0.212*	−0.0594	0.090	−0.002	−0.047	−0.019	−0.107	−0.008	−0.048	−0.074	−0.083
Vegetable group adequacy	0.067	0.081	0.111*	0.001	−0.068	0.132	0.073	0.197**	0.041	0.029	0.006	0.096	0.094	0.138	0.117
Fruit group adequacy	0.020	−0.02	0.051	0.070	−0.080	0.043	−0.058	0.018	0.028	0.133	−0.006	0.001	−0.061	−0.032	0.074
Grain group adequacy	0.109	0.266**	−0.011	0.150	0.218*	0.032	0.113	0.324**	−0.006	0.017	0.221*	−0.019	0.169*	0.296**	−0.002
Fiber adequacy	0.138*	0.150**	0.072	0.186*	0.112	−0.006	0.114	0.181*	0.138	0.164	0.027	−0.040	0.118	0.235**	0.136
Iron adequacy	0.013	0.069	−0.048	0.140	0.185*	−0.023	0.032	0.058	0.003	0.034	0.024	−0.104	−0.007	0.093	−0.019
Calcium adequacy	0.018	0.054	−0.118*	0.098	0.049	−0.066	0.035	0.105	-0.092	0.102	0.106	−0.083	−0.023	0.025	−0.110
Vitamin C adequacy	0.159**	0.091	0.103	0.169*	0.007	0.101	0.091	0.133	0.025	0.236**	0.045	0.093	0.108	0.128	0.107
Total adequacy	0.133*	0.173**	0.034	0.222**	0.101	0.086	0.089	0.237**	0.013	0.181*	0.092	−0.031	0.105	0.226**	0.077
Total fat moderation	0.015	−0.031	−0.019	0.139	0.052	0.041	−0.099	−0.105	−0.097	0.100	−0.036	−0.011	−0.040	-0.019	-0.018
Saturated fat moderation	0.051	−0.032	0.021	0.054	-0.036	0.047	0.018	−0.048	−0.039	0.024	−0.078	0.014	0.070	0.010	0.037
Cholesterol moderation	0.015	−0.086	0.096	−0.043	−0.167	0.067	−0.026	−0.069	0.047	−0.057	−0.079	0.156	0.062	−0.084	0.075
Sodium moderation	0.004	−0.166**	0.091	0.001	−0.063	0.126	−0.077	−0.291**	0.003	−0.042	−0.157	0.107	0.036	−0.167*	0.100
Empty calories food moderation	−0.111*	−0.173**	0.047	−0.113	−0.179*	0.169*	−0.129	−0.175*	−0.056	−0.045	−0.119	0.006	−0.155*	−0.206**	0.070
Total moderation	−0.003	−0.151**	0.084	−0.015	−0.121	0.131	−0.104	−0.225**	−0.032	−0.014	−0.149	0.112	0.011	−0.139	0.087
Macronutrient ratio	0.094	0.096	0.031	0.137	0.106	0.041	0.044	0.086	−0.006	0.052	0.089	0.035	0.114	0.100	0.023
Fatty acid ratio	−0.078	−0.167**	0.021	−0.179*	−0.269**	0.039	0.036	−0.068	0.041	−0.098	−0.129	0.043	−0.061	−0.193**	0.004
Overall balance	−0.036	−0.113*	0.032	−0.160	−0.247**	0.056	0.056	−0.010	0.023	−0.069	−0.084	0.060	−0.010	−0.130	0.012
Total DQI-I	0.057	−0.059	0.080	0.018	−0.161	0.145	0.029	−0.004	−0.028	0.090	−0.087	0.089	0.038	−0.018	0.085

Lowest correlation coefficient r — Midpoint correlation coefficient r — Highest correlation coefficient r

Figure S1. Heat map of correlation between eating behavior and diet quality. Correlation coefficient r is presented in the square and the strength of correlation is depicted in color, with red being the lowest and blue the highest correlation coefficient: * $p < 0.05$, ** $p < 0.01$. EmoE - Emotional eating, ExtE - External Eating, RE - Restrained Eating, DQI-I – Diet Quality Index–International.

Table S1. Regression analysis of the associations between eating behavior and diet quality for the crude model

DQI-I component	EmoE				ExtE				RE			
	β	95%CI	Partial R^2	*p*-value	β	95%CI	Partial R^2	*p*-value	β	95%CI	Partial R^2	*p*-value
**Variety**	0.001	-0.238-0.243	0.001	0.983	-0.079	-0.435-0.074	0.079	0.164	-0.015	-0.300-0.230	0.015	0.794
Food groups	-0.008	-0.194 – 0.167	0.008	0.886	-0.100	-0.361-0.019	0.100	0.078	-0.028	-0.248-0.149	0.028	0.149
Protein sources	0.017	-0.088-0.119	0.017	0.765	-0.010	-0.119-0.100	0.010	0.864	0.014	-0.100-0.128	0.014	0.808
**Adequacy**	0.084	-0.127-0.907	0.084	0.139	0.174	0.316-1.399	0.174	**0.002**	0.012	-0.509-0.634	0.012	0.829
Vegetable group	0.039	-0.077-0.160	0.039	0.493	0.076	-0.040-0.210	0.076	0.181	0.077	-0.040-0.220	0.077	0.172
Fruit group	0.014	-0.113-0.146	0.014	0.800	-0.028	-0.172-0.102	0.028	0.616	0.100	-0.014-0.270	0.100	0.076
Grain group	0.141	0.037-0.302	0.141	**0.013**	0.280	0.221--0.494	0.280	**< 0.001**	-0.043	-0.204-0.091	0.043	0.452
Fiber	0.097	-0.015-0.235	0.097	0.085	0.167	0.069-0.331	0.167	**0.003**	0.061	-0.063-0.213	0.061	0.285
Iron	-0.050	-0.155-0.059	0.050	0.375	0.042	-0.070-0.156	0.042	0.453	-0.106	-0.229-0.005	0.106	0.061
Calcium	-0.044	-0.233-0.100	0.044	0.434	0.045	-0.105-0.248	0.045	0.425	-0.131	-0.398--0.034	0.131	**0.020**
Vitamin C	0.133	0.029-0.305	0.133	**0.018**	0.102	-0.012-0.282	0.102	0.071	0.111	0.000-0.306	0.111	**0.049**
**Moderation**	-0.024	-0.833-0.538	0.024	0.672	-0.150	-1.699--0.262	0.150	**0.008**	0.075	-0.243-1.263	0.075	0.184
Total fat	-0.036	-0.223-0.113	0.036	0.521	-0.023	-0.216-0.141	0.023	0.680	-0.026	-0.228-0.143	0.026	0.652
Saturated fat	0.030	-0.146-0.255	0.030	0.593	-0.047	-0.302-0.123	0.047	0.409	-0.001	-0.223-0.219	0.001	0.984
Cholesterol	0.008	-0.216-0.252	0.008	0.882	-0.087	-0.441-0.053	0.087	0.124	0.080	-0.072-0.442	0.080	0.158
Sodium	0.037	-0.152-0.301	0.037	0.518	-0.154	-0.569--0.095	0.154	**0.006**	0.103	-0.018-0.479	0.103	0.068
Empty calories foods	-0.128	-0.447—0.032	0.128	**0.024**	-0.165	-0.546--0.109	0.165	**0.003**	0.067	-0.091-0.368	0.067	0.235
**Overall balance**	0.013	-0.122-0.153	0.013	0.825	-0.102	-0.279-0.012	0.102	0.072	0.024	-0.119-0.185	0.024	0.669
Macronutrient ratio	0.064	-0.029-0.108	0.064	0.260	0.099	-0.008-0.137	0.099	0.080	0.002	-0.075-0.077	0.002	0.979
Fatty acid ratio	-0.022	-0.144-0.096	0.022	0.696	-0.173	-0.323—0.073	0.173	**0.002**	0.027	-0.100-0.164	0.027	0.634
**Total DQI-I score**	0.036	-0.549-1.070	0.036	0.527	-0.057	-1.293-0.420	0.057	0.317	0.071	-0.319-1.459	0.071	0.208

DQI-I – diet quality index-international, β - standardized coefficients, CI – confidence interval, R2 – coefficient of determination, EmoE - Emotional eating. DQI-I – Diet quality index-International. Linear regression model and enter method were used. Crude model – model examining the unadjusted association between eating behavior and diet quality. Bold values denote statistical significance at the $p < 0.05$ level.

Table S2. Regression analysis of the associations between eating behavior and diet quality for Model 2

	EmoE				ExtE				RE			
**DQI-I component**	**β**	**95%CI**	**Partial R^2**	***p*-value**	**β**	**95%CI**	**Partial R^2**	***p*-value**	**β**	**95%CI**	**Partial R^2**	***p*-value**
**Variety**	-0.009	-0.282-0.241	-0.009	0.878	-0.098	-0.495-0.045	-0.001	0.102	-0.019	-0.326-0.236	-0.009	0.754
Food groups	-0.025	-0.236-0.155	-0.006	0.682	-0.118	-0.404--0.001	0.006	0.049	-0.041	-0.283-0.137	0.020	0.496
Protein sources	0.022	-0.092-0.133	-0.015	0.723	-0.023	-0.139-0.095	-0.015	0.708	0.027	-0.093 0.149	-0015	0.650
**Adequacy**	0.035	-0.347-0.677	0.165	0.526	0.090	-0.085-0.974	0.171	0.100	0.052	-0.286-0.815	0.166	0.345
Vegetable group	0.021	-0.105-0.149	0.018	0.733	0.067	-0.056-0.207	0.022	0.257	0.050	-0.077-0.195	0.020	0.395
Fruit group	0.000	-0.139-0.139	0.011	0.999	-0.039	-0.193-0.096	0.012	0.508	0.110	-0.008-0.289	0.022	0.063
Grain group	0.101	-0.015-0.258	0.107	0.081	0.213	0.132-0.411	0.139	0.000	-0.038	-0.198-0.098	0.099	0.505
Fiber	0.052	-0.067-0.185	0.138	0.355	0.087	-0.027-0.234	0.143	0.118	0.083	-0.033-0.238	0.142	0.137
Iron	-0.022	-0.125-0.082	0.197	0.682	0.030	-0.077-0.138	0.197	0.581	-0.004	-0.116-0.107	0.196	0.942
Calcium	-0.058	-0.240-0.065	0.282	0.260	-0.023	-0.195-0.122	0.280	0.655	-0.070	-0.279-0.048	0.284	0.166
Vitamin C	0.057	-0.073-0.215	0.081	0.333	0.036	-0.102-0.198	0.080	0.531	0.096	-0.023-0.287	0.087	0.094
**Moderation**	0.029	-0.397-0.749	0.401	0.546	-0.052	-0.937-0.252	0.403	0.258	0.026	-0.442-0.791	0.401	0.579
Total fat	-0.029	-0.219-0.131	0.072	0.620	0.017	-0.154-0.209	0.072	0.766	-0.058	-0.285-0.091	0.074	0.310
Saturated fat	0.071	-0.078-0.332	0.105	0.223	0.010	-0.195-0.232	0.123	0.866	-0.019	-0.259-0.183	0.101	0.737
Cholesterol	0.054	-0.091-0.320	0.340	0.273	0.002	-0.209-0.218	0.337	0.966	0.034	-0.141-0.301	0.338	0.479
Sodium	0.073	-0.040-0.338	0.401	0.122	-0.064	-0.335-0.058	0.400	0.167	0.035	-0.127-0.282	0.398	0.456
Empty calories foods	-0.091	-0.391-0.050	0.045	0.129	-0.128	-0.482—0.026	0.053	0.029	0.074	-0.085-0.390	0.043	0.209
**Overall balance**	0.045	-0.093-0.205	-0.004	0.463	-0.083	-0.264-0.045	0.001	0.165	0.019	-0.135-0.186	-0.005	0.754
Macronutrient ratio	0.028	-0.057-0.091	0.000	0.651	0.079	-0.025-0.129	0.005	0.186	-0.040	-0.107-0.053	0.001	0.503
Fatty acid ratio	0.036	-0.090-0.167	0.019	0.554	-0.141	-0.293—0.029	0.036	0.017	0.044	-0.085-0.191	0.020	0.453
**Total DQI-I score**	0.052	-0.460-1.213	0.084	0.376	-0.030	-1.102-0.637	0.083	0.599	0.052	-0.480-1.320	0.084	0.360

DQI-I – diet quality index-international, β - standardized coefficients, CI – confidence interval, R2 – coefficient of determination, EmoE - Emotional eating. DQI-I – Diet quality index-International. Linear regression model and enter method were used. Model 2 –adjusted for sex, age, duration of diabetes, area of residence, employee status, daily energy intake and physical activity level. Bold values denote statistical significance at the $p < 0.05$ level.
